# Predicting the trabecular bone apparent stiffness tensor with spherical convolutional neural networks

**DOI:** 10.1016/j.bonr.2022.101179

**Published:** 2022-03-07

**Authors:** Fabian Sinzinger, Jelle van Kerkvoorde, Dieter H. Pahr, Rodrigo Moreno

**Affiliations:** a KTH Royal Institute of Technology, Department of Biomedical Engineering and Health Systems, Sweden; b Eindhoven University of Technology, the Netherlands; c Technical University of Vienna, Institute for Lightweight Design and Structural Biomechanics, Austria; d Karl-Landsteiner University, Biomechanics Division, Austria

**Keywords:** Apparent stiffness tensor, Trabecular bone, Spherical convolutional neural networks, SphCNN, Spherical convolutional neural networks, EGI, Extended Gaussian image, Tb.Th, Trabecular thickness, Tb.Sp, Trabecular spacing

## Abstract

The apparent stiffness tensor is relevant for characterizing trabecular bone quality. Previous studies have used morphology-stiffness relationships for estimating the apparent stiffness tensor. In this paper, we propose to train *spherical convolutional neural networks* (SphCNNs) to estimate this tensor. Information of the edges, trabecular thickness, and spacing are summarized in functions on the unitary sphere used as inputs for the SphCNNs. The concomitant dimensionality reduction makes it possible to train neural networks on relatively small datasets. The predicted tensors were compared to the stiffness tensors computed by using the *micro-finite element method* (μFE), which was considered as the gold standard, and models based on fourth-order fabric tensors. Combining edges and trabecular thickness yields significant improvements in the accuracy compared to the methods based on fourth-order fabric tensors. From the results, SphCNNs are promising for replacing the more expensive μFE stiffness estimations.

## Highlights

• *Characteristic stiffness tensors* are derived from trabecular bone micro-CT samples.
• Previous approximation methods fall short on heterogeneous data-sets.
• The *gradient*, *trabecular thickness* and *spacing* are mapped to a *spherical domain*.
• *Spherical convolutional neural networks* are used for the prediction.
• The prediction error is significantly reduced compared to the state-of-the-art.

## Graphical abstract

**Figure:**
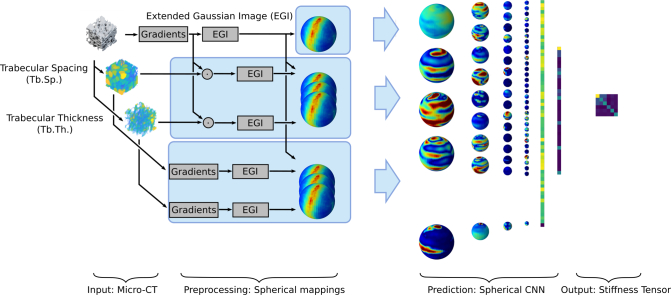

## 1 Introduction

*Osteoporosis* is a disorder that negatively affects the composition and architecture of bone tissue, which can decrease bone mass and increase skeletal fragility (Boutroy et al., 2005). The stage of osteoporosis is commonly diagnosed using *areal bone mineral density* (aBMD) as measured by *dual-energy x-ray absorptiometry* (DXA), but its accuracy is limited mainly due to its 2D nature (Miller et al., 2002; Marshall et al., 1996). Many studies have found that the mechanical properties of trabecular bone tissue could be used to distinguish between osteoporotic and non-osteoporotic bone. Thus, bone mechanics can be used to improve medical diagnosis, surgical planning, intervention, and fracture risk assessment (Kim et al., 2014; Tjhia et al., 2011, Tjhia et al., 2012; Nyman et al., 2016). One of the mechanical properties that is often used for this purpose is bone stiffness (Steiner et al., 2017; Ovesy et al., 2020). Stiffness properties are commonly described by the apparent stiffness tensor, a mathematical entity that relates stress and strain of the microarchitecture of the tissue.

There are several approaches for estimating the apparent stiffness tensor. For example, mechanical tests can be performed on in vitro samples. However, the estimation of the apparent stiffness tensor using this method can only be partially done as every test can destroy the tissue. A better alternative is to use *finite element* (FE) modeling with appropriate boundary conditions (Pahr and Zysset, 2008). Using FE modeling is advantageous since it is able to estimate the full apparent stiffness tensor without the drawbacks of mechanical testing. However, the main drawback of FE modeling is that it is computationally expensive.

An alternative to FE is to approximate the apparent stiffness tensor by assuming a close relationship between morphology and stiffness. For that, the apparent stiffness tensor is modeled as a function of mechanical properties of the matrix, bone density, and fabric tensors (Zysset, 2003; Moreno et al., 2016). The main advantage of this approach is that the computational cost is negligible, which makes it appealing for clinical applications where time might become an issue. On the contrary, this approach is less accurate than FE. For example, the model by Moreno et al. (2016) is still far from ideal, with 30–40% error in some cases.

In recent years, machine learning techniques, and in particular *deep learning* (DL), have gained popularity to solve problems in the field of biomechanics. For example, Xiao et al. (2020) trained a neural network to predict histomorphometric parameters from simulated DXA images. Moreover, Nissinen et al. (2021) used DL to classify DXA images. DL has also been used for bone classification (Shen et al., 2021; Tanzi et al., 2020). Regarding the estimation of biomechanical parameters, different groups have used DL for approximating biomechanical properties of vessel walls using FE models as gold standard (Liang et al., 2018; Madani et al., 2019; Rengarajan et al., 2021). These results make DL appealing to be used for predicting the apparent stiffness tensor in trabecular bone, which, to our knowledge, has not been tested so far by other research groups.

The main drawback of using DL in any application is that it typically requires a lot of training data, which is a big challenge for stiffness estimation. Here, the implementation of an end-to-end solution with *convolutional neural networks* (CNNs) with 3D volumes of trabecular bone as input and the entries of the apparent stiffness tensor as output might require hundreds of thousands of training samples due to the high number of parameters to be trained in the neural network. Rather than using transfer-learning for the domain adaptation of such a high-parametric model, we suggest an alternative strategy to tackle this problem.

Instead of the original image of trabecular bone, we propose using the *extended Gaussian images* (EGI) as the input of the neural network. The EGI is a function on the unitary sphere *S*^2^ that captures the orientation distribution of the gradient of the image. Since the EGI is a function on *S*^2^, it is suitable to use *spherical convolutional neural networks* (SphCNNs) (Esteves et al., 2020; Cohen et al., 2018) instead of standard CNNs since the latter requires data on cartesian grids. The main advantage of using SphCNNs here is that it is possible to reduce the number of parameters to learn vastly. Concretely, the SphCNN of this study has about 0.4 M trainable parameters, whereas the 3D version of the famous VGG16 consists of over 170 M parameters as reported in (Leong et al., 2020).

While the EGI conveys the edge information of trabecular bone, it completely disregards the distribution of bone material in the volume. To some extent, that information is captured by the *trabecular thickness* (Tb.Th) and *trabecular spacing* (Tb.Sp). Thus, we also propose to combine EGI with Tb.Th and Tb.Sp in order to improve the accuracy of the neural network for stiffness prediction.

### 1.1 Contributions

The main contributions of this paper are:

• To the best of our knowledge, this is the first attempt to use DL, specifically SphCNNs, to estimate the apparent stiffness tensor. The results show that DL is more accurate than fabric tensor-based approaches.
• A novel way of combining edge information encoded in the EGI with Tb.Th and Tb.Sp is proposed, that is meaningful for DL models.

## 2 Material

The present study utilized the data from Gross et al. (2013), which was also partially used in Moreno et al. (2016). For a more detailed description, we refer here to the respective reports. A total of 700 gray-level images of trabecular bone cubes were obtained by scanning three proximal femora, three distal radii, and six vertebral bodies with μ-CT (μ-CT 40, SCANCO Medical AG, Brüttisellen, Switzerland). In total, we used 355 cubes from the vertebra, 264 from the femur 81 from the radius. All scans were taken at 18 μm isotropic resolution and further processed by applying a 3D Gaussian filter (*σ* = 1.2, support = 2). After dividing each specimen into the cubic subregions with a side length of 5.3 mm, the volumes were segmented via the application of a single-level threshold of IPL (SCANCO Medical AG, Brüttisellen, Switzerland). Furthermore, for each subsection, structures that were not connected to the main region after thresholding were removed from the volumes. Subsequently, each subregion was transferred into a piece-wise linear domain by considering each voxel as a hexahedral eight-node element. The Young modulus and the Poisson's ratio assigned to each element were 12 GPa and 0.3, respectively. Finally, FE-simulations were executed for six different loading scenarios under kinematic boundary conditions as described in Pahr and Zysset (2008) to obtain the full apparent stiffness tensor computed through strain and stress averages. FE simulations were performed using Abaqus (Dassault Systmes, Paris, France), and the apparent stiffness tensor of each bone cube was computed from the results of the six load steps by using Medtool (http://www.dr-pahr.at).

## 3 Methods

### 3.1 Apparent stiffness tensor

In general, the *apparent stiffness tensor C* of a material describes its mechanical properties by relating its strain (*ϵ*) and stress tensors (*σ*) through the Hooke's law:

(1) $$\sigma_{\mathrm{ij}}=C_{\mathrm{ijk}\ell }\epsilon_{k\ell }\cdot$$

The apparent stiffness tensor is represented as a fourth-order tensor with 3 × 3 × 3 × 3 = 81 coefficients. However, symmetry constraints reduce the number of independent entries of the apparent stiffness tensor to 21 (Moreno et al., 2016). Alternatively, apparent stiffness tensors can be represented as symmetric 6 × 6 matrices (Moreno et al., 2016). In this paper, we modeled the apparent stiffness tensor as a vector with 36 entries.

### 3.2 Trabecular thickness and spacing

As mentioned, we wanted to explicitly expose the DL model to information about the local thickness of trabeculae. Furthermore, we hypothesized that the spacing between the trabecular structures contains valuable information for predicting the mechanical properties. Let *p* be a point in the image. Local thickness (Tb.Th) at that point is derived according to (Hildebrand and Rüegsegger, 1997):

(2) $$\mathrm{Tb}.\mathrm{Th}\left(p\right)=2\max\left(\left\{r\left|\mathrm{p\epsilon}B_{r}\left(t\right)\forall B_{r}\left(t\right)\subseteq T\right)\right\}\right),$$

with *T* being the segmented trabecular bone, *B*_*r*_(*t*) is a ball centered at *t* with radius *r*, that lies completely inside the trabecular bone. Tb.Th is zero for points outside the trabecular bone. Local spacing Tb.Sp is defined analogously for points outside the trabecular bone:

(3) $$\mathrm{Tb}.\mathrm{Sp}\left(p\right)=2\max\left(\left\{r\left|\mathrm{p\epsilon}B_{r}\left(t\right)\forall B_{r}\left(t\right)\subseteq \overset{ˇ}{T}\right)\right\}\right),$$

with $\overset{ˇ}{T}$ being the complement of *T*, and Tb. Sp(*p*) = 0 for *pϵT*.

**Fig. 1:**
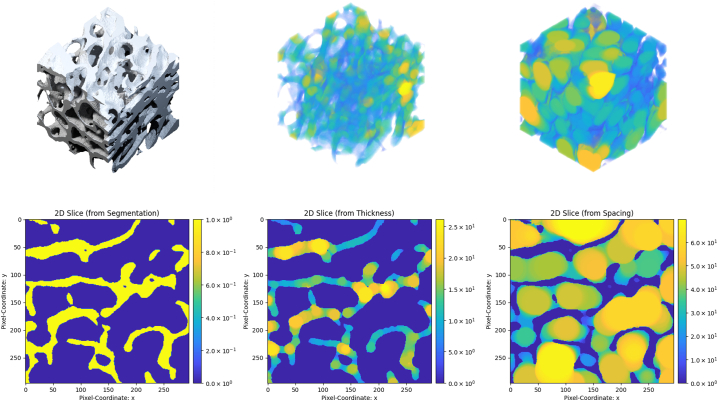
Top row: Graphical representations of the initial μ-CT trabecular segmentation (left), the trabecular thickness (middle), and trabecular spacing (right). Bottom row: central slices of the respective three-dimensional pixel arrays (segmentation: left, thickness: middle, spacing: right).

Fig. 1 shows the segmentation, thickness, and spacing maps of one of the tested specimens.

Notice that we use local maps of Tb.Th and Tb.Sp instead of the customary use of the mean in the analysis of bone specimens, e.g., Guha et al. (2020); Steiner et al. (2020). The thickness and spacing maps used in the experiments were computed with BoneJ (Doube et al., 2010).

### 3.3 Extended Gaussian images

Information about the orientation of the surfaces of an object can be obtained by mapping the surface normal onto a unit sphere. This way of mapping the surface normals is called an *extended Gaussian image* (EGI). The surface normals are mapped onto the sphere by putting their tails at the center of the Gaussian sphere and their heads at the appropriate place on the surface of the Gaussian sphere (Horn, 1984).

For a segmented image *x*, it is possible to estimate the EGI from the gradient of the image for a specific direction *v* as Moreno et al. (2012b):

(4) $$\mathrm{EGI}\left(v\right)=\int_{s^{2}}\delta \left(\langle \frac{\nabla x}{\left\|\nabla x\right\|+\epsilon },v\rangle -1\right)\left\|\nabla x\right\|\mathrm{ds},$$

with *δ*(·) being the unit impulse function, < · , · > the dot product, and *ϵ* a small constant to avoid the division by zero. Thus, the EGI can be seen as the orientation distribution function of the gradient of the image. Since the gradient of *x* can only take values of zero or infinity, it is customary to perform a Gaussian smoothing with a very small standard deviation before the gradients are calculated.

**Fig. 2:**
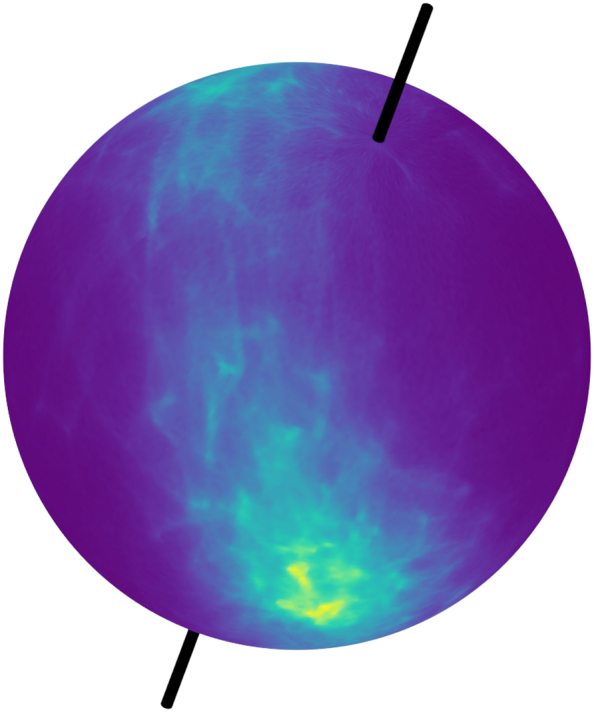
Extended Gaussian Image (EGI) displayed on 3D sphere mesh with a stick (black) through the poles. Brighter regions of the surface correspond to higher values in the EGI.

**Fig. 3:**
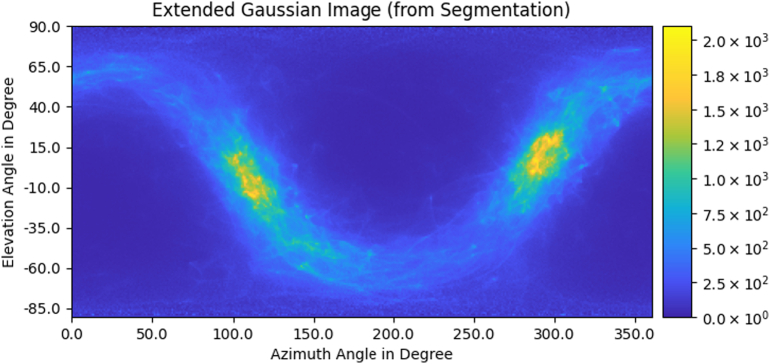
Extended Gaussian Image (EGI) unwrapped on the *α*-*β*-plane.

In this paper, the EGI is parameterized by the spherical coordinates (*α*, *β*) with *αϵ*[0, 2*π*] being the azimuth *βϵ*[0, *π*] being the elevation. One example of a resulting EGI on a sphere and unwrapped on the *α*-*β*-plane is visualized in Fig. 2 and Fig. 3, respectively.

Notice that the EGI can be used to compute different fabric tensors, including the *mean intercept length* (MIL) tensor, the *generalized MIL* (GMIL) tensor, and the *global structure tensor* (GST) (Moreno et al., 2016). Thus, the computation of the EGI is also necessary for estimating stiffness from bone morphology.

### 3.4 Combining the gradient with thickness and spacing

As already mentioned, the EGI contained the edge information of trabecular bone. In order to consider the distribution of bone and marrow material in the image, Tb.Th and Tb.Sp maps are combined with the EGI.

The first idea is to use the Tb.Th and Tb.Sp maps as weighting functions in the estimation of the EGI:

(5) $$\mathrm{EGI}\left(g,v\right)=\int_{s^{2}}\mathrm{g\delta}\left(\langle \frac{\nabla x}{\left\|\nabla x\right\|+\epsilon },v\rangle -1\right)\left\|\nabla x\right\|\mathrm{ds},$$

with *g* being either Tb.Th or Tb.Sp maps. Since the gradient is non-zero only in the interface between bone and marrow, the effect of this weighting is to create an orientation distribution function of the trabecular thickness (or spacing). Thus, the regions with the thickest trabeculae (or marrow) will contribute more to the EGI.

**Fig. 4:**
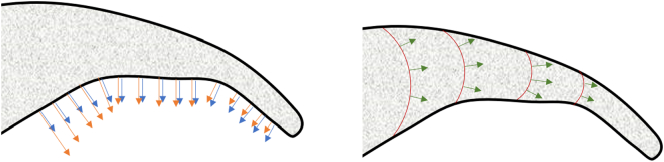
Strategies for combining the gradient and trabecular thickness. Left: the gradient of trabecular bone (blue arrows) is used to create the EGI. Then, it is scaled with the local thickness information (orange arrows) in order to create an additional EGI. Notice that scaling is only performed on the surface since the gradient is zero elsewhere. Right: the gradient of the thickness map (green arrows) is used to create an additional EGI. Isosurfaces of trabecular thickness are shown in red.

Alternatively, one can create EGIs by using the Tb.Th and Tb.Sp maps instead of the segmented image. In this case, the EGI is computed directly from the gradient of Tb.Th and Tb.Sp using Eq. (4). The information encoded by these EGIs is related to the local variability of the size of the trabeculae (or marrow), which can convey relevant information for stiffness estimations. Fig. 4 shows a scheme of these two approaches for the case of Tb.Th.

### 3.5 Spherical CNNs

In our case, the input of our neural network are signals on the *2-sphere* (*S*^2^). Thus instead of traditional CNNs, we opted to use SphCNNs (Esteves et al., 2020).

The main difference between CNNs and SphCNNs is the way the convolution filters are structured. While conventional CNN filters operate on a gridded data structure, in SphCNNs, the convolution filters are zonal kernels on *S*^2^. Convolutions on *S*^2^ are rendered by a pointwise multiplication in the spherical harmonics domain of the spherical input and the zonal filters.

The spherical harmonics transform of a function *f* on *S*^2^, and its inverse are given by (Arfken et al., 2013; Driscoll and Healy, 1994):

(6) $${\hat{f}}_{m}^{l}=\int_{s^{2}}f\left(x\right)Y_{m}^{l}\mathrm{dx},$$

and

(7) $$f=\sum_{0\leq l\leq b}\sum_{\left|m\right|\leq l}{\hat{f}}_{m}^{l}Y_{m}^{l},$$

respectively, with *b* being the bandwidth of *f* and *Y*_*m*_^*l*^ are the spherical harmonics of degree *l* and order *m*.

The convolution of *f* with a zonal kernel *h* can be computed as (Esteves et al., 2020):

(8) $${\hat{y}}_{m}^{l}=2\pi \sqrt{\frac{4\pi }{2l+1}{\hat{f}}_{m}^{l}{\hat{h}}_{0}^{l},}$$

where ${\hat{y}}_{m}^{l}$ is the spherical harmonics transform of *f* ∗ *h*. Thus, convolutions can be implemented very efficiently in the spherical harmonics domain.

After the last convolution layer, a weighted global average pooling is applied in which the difference in the area of the different areas of the sampling of the *S*^2^ is taken into account. Further details of SphCNN can be found in Esteves et al. (2020).

### 3.6 SphCNNs for predicting the apparent stiffness tensor

**Fig. 5:**
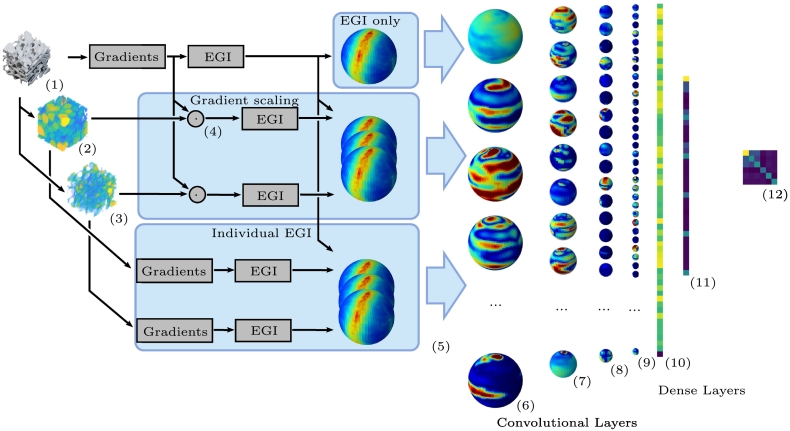
Overview of the proposed prediction pipeline. Given the segmentation of trabecular bone (1), local maps of Tb.Sp (2), Tb.Th (3) are computed. Using the gradient of the (smoothed) segmented image, we compute the EGI. Tb.Th and Tb.Sp are used independently to weight the gradient of the image (4). We also compute the gradients of Tb.Th and Tb.Sp for computing their EGI. We trained different SphCNNs with different inputs (5). The first type of input consists of the original EGI (top arrow). The second type combines the EGI with the weighted EGIs (middle arrow) as seperate channels. The third type of input includes the EGI together with the EGI of Tb.Th and Tb.Sp (bottom arrow). Each SphCNN is composed of spherical convolutional layers and fully connected layers that generate feature maps (6–11). Finally, the output of the last layer is reshaped as a 6 × 6 matrix that represents the predicted apparent stiffness tensor (12).

Fig. 5 shows the proposed pipeline for the estimation of the apparent stiffness tensor. SphCNNs are trained using three different configurations. In the first one, the SphCNN receives only the EGI as an input, in the second one, two extra channels are added with the weighted EGIs computed through Eq. (5), and in the third two extra channels with the EGI of Tb.Th and Tb.Sp were included. As aforementioned, we expect that providing information about the distribution of bone and marrow material can lead to improved estimations of the stiffness.

## 4 Experimental results

### 4.1 Model architecture

#### 4.1.1 Model input

The input of the neural network consists of one or multiple EGIs. In the first experiments, only the EGI of the segmentation is used. In further experiments, we add EGIs that contain thickness and spacing information as additional channels of the same size. Every EGI is represented as a 2D pixel array of size 64× 64.

#### 4.1.2 Spherical convolutional layers

The activation signals of the SphCNN pass a stack of three spherical convolutional layers with 64, 128, and 256 convolutional filters, respectively. Each filter is parameterized by 32 coefficients. Weighted average pooling layers are added after each spherical convolutional layer to reduce the size of the feature maps by a factor of two. Here, the implementation of the spherical convolutional layers by Esteves et al. (2020)^1^ was used.

#### 4.1.3 Dense and output layers

After the convolutional stack, we average the activations per channel resulting in a 1D signal of size 256. This signal is fed to a stack of two dense layers that reduce the size first to 64 and then to 36. These 36 entries represent the components of the 6 × 6 stiffness matrix. The last output layer of this model does not use any nonlinearity (i.e., an identity mapping is used as activation function). This enables us to output the continuous stiffness values. All other layers employ *parametric rectified linear units* (PRELU) (He et al., 2015) as nonlinearities.

#### 4.1.4 Training routine

All experiments described in this report share the same training configuration (see Table 1). These hyperparameters were determined empirically. In addition, experiments were run with 5-fold cross-validation.

**Table 1:** Configuration of the SphCNN training pipeline used for the experiments.

Batch-size	16
Steps per epoch	One sweep over the respective training set, reshuffled every epoch
Number of epochs	600
Learning-rate	Epoch 0–199: 0.01, Epoch 200–399: 0.001, Epoch 400–599: 0.001.
Optimizer	ADAM
Dropout	No
Batch-normalization	Yes
Loss	Frobenius Error

The Frobenius error serves as both, the performance metric and the loss function. It is defined by:

(9) $$E_{F}=\frac{{\left\|C_{o}-C_{p}\right\|}_{F}}{{\left\|C_{o}\right\|}_{F}},$$

where *C*_*o*_ and *C*_*p*_ are the observed and predicted apparent stiffness tensors and ‖·‖_*F*_ is the Frobenius norm.

### 4.2 Comparison of different architectures

In Table 2, three input-combination candidates described in Section 3.4 were compared and evaluated on the individual skeletal sites. Noticeably, adding thickness and scaling with either method led to a significantly lower Frobenius error across all evaluated regions. As shown, including the gradients of the thickness and spacing maps (‘Channels’ in Table 2) was slightly superior to the scaling option (‘Scaling’ in Table 2) with respect to their predictive performance. Thus, the errors are reduced between 7 and 11% depending on the skeletal site for the ‘Channels’ strategy compared to the use of the EGI alone. Based on these findings, we speculate that our projection into the spherical domain dropped information that can be partially re-introduced with the proposed addition of local thickness and spacing. Moreover, the accuracy is relatively insensitive to the skeletal site.

**Table 2:** Mean Frobenius Errors of the models trained with different feature channels and evaluated on different bone-regions. ‘EGI’ means that only the Extended Gaussian Image from the initial segmentation is used. ‘Channels’ refers to the combination method where the gradients and subsequent EGI were computed from the Tb.Th and Tb.Sp maps directly. ‘Scaling’ refers to the inclusion of two additional channels computed from gradients weighted with Tb.Th and Tb.Sp, respectively. The best average performance (i.e., lowest observed errors) is highlighted in bold.

		Evaluation region			
Input	Fold	All	Radius	Vertebra	Femur
EGI	1	0.35	0.29	0.40	0.30
	2	0.33	0.27	0.38	0.27
	3	0.29	0.27	0.26	0.34
	4	0.28	0.21	0.30	0.28
	5	0.30	0.31	0.30	0.31
	Mean	0.31	0.27	0.33	0.30
Channels	1	0.24	0.22	0.25	**0.23**
	2	0.21	0.14	0.23	0.21
	3	0.23	0.18	0.23	0.26
	4	0.22	0.18	0.22	0.24
	5	0.21	0.30	0.19	0.21
	Mean	**0**.**22**	**0**.**20**	**0**.**22**	**0**.**23**
Scaling	1	0.21	0.17	0.21	0.24
	2	0.25	0.26	0.24	0.25
	3	0.20	0.18	0.22	0.18
	4	0.24	0.22	0.23	0.25
	5	0.23	0.24	0.21	0.26
	Mean	0.23	0.22	**0.22**	0.24

**Fig. 6:**
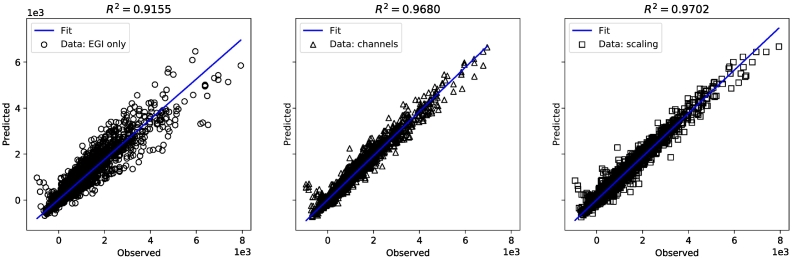
Correlation plots of the 36 terms of the observed versus the predicted 6 × 6 stiffness tensors for the three feature combination methods: ‘EGI’, ‘Channels’, ‘Scaling’ on the complete dataset.

To further support the assumption that the additional feature channels are beneficial, we analyze the correlation of the predicted and observed tensors' entries. Fig. 6 shows correlation plots of the entries for the respective combination methods. As shown, *R*^2^ is lower when using only the EGI for prediction than the experiments where additional thickness and spacing local maps were incorporated (5–6% improvement in *R*^2^), with the scaling option being slightly better.

In Table 3, we also included experiments where Tb.Th or Tb.Sp are disregarded from the ‘Channels’ and ‘Spacing’ setups (cf. ‘Scaling (Tb.Sp)’, ‘Scaling (Tb.Sp)’, ‘Scaling (Tb.Sp)’, and ‘Scaling (Tb.Sp)’ experiments in the table). These experiments aimed to assess which of these two features contributed more to the accuracy of the predictions. Compared to the use of EGI alone, including Tb.Th leads to a reduction of the error between 7 and 13% for the ‘Channels’ strategy, depending on the skeletal site. These results outperform the ones from Table 2, which means that including Tb.Sp can have a negative impact on the predictive power of the SphCNN. In fact, the performance of the ‘Channels’ strategy with only Tb.Sp is worse than the sole use of the EGI. Multiple pairwise tests (cf. Table A.11) showed however that this difference in performance was not significant. Table 2 also shows that Tb.Th is more relevant than Tb.Sp for the ‘Scaling’ strategy. However, unlike the ‘Channels’ strategy, adding both Tb.Th and Tb.Sp is beneficial for reducing the error of the predictions.

**Table 3:** Mean Frobenius Errors of the Models trained with different feature channels and evaluated on different bone-regions. ‘Channels (Tb.Sp)’ and ‘Channels (Tb.Th)’ use the same strategy of ‘Channels’ (see Table 2) but disregard Tb.Th and Tb.Sp, respectively. ‘Scaling (Tb.Sp)’ and ‘Scaling (Tb.Th)’ use the same strategy of ‘Scaling’ (see Table 2) but disregard Tb.Th and Tb.Sp, respectively. The best average performance (i.e., lowest observed errors) is highlighted in bold.

		Evaluation region			
Input	Fold	All	Radius	Vertebra	Femur
Channels	1	0.33	0.25	0.31	0.39
(Tb.Sp)	2	0.35	0.27	0.39	0.34
	3	0.30	0.23	0.34	0.28
	4	0.32	0.51	0.28	0.32
	5	0.33	0.34	0.30	0.37
	Mean	0.33	0.32	0.33	0.34
Channels	1	0.21	0.20	0.21	0.22
(Tb.Th)	2	0.21	0.19	0.22	0.20
	3	0.19	0.19	0.18	0.19
	4	0.21	0.20	0.21	0.22
	5	0.20	0.21	0.19	0.21
	Mean	**0**.**20**	**0**.**20**	**0**.**20**	**0**.**21**
Scaling	1	0.25	0.19	0.25	0.28
(Tb.Sp)	2	0.27	0.23	0.28	0.28
	3	0.26	0.19	0.31	0.23
	4	0.28	0.32	0.28	0.26
	5	0.33	0.33	0.33	0.35
	Mean	0.28	0.25	0.29	0.28
Scaling	1	0.24	0.23	0.23	0.26
(Tb.Th)	2	0.25	0.22	0.25	0.25
	3	0.23	0.21	0.24	0.21
	4	0.26	0.29	0.27	0.26
	5	0.28	0.25	0.28	0.29
	Mean	0.25	0.24	0.25	0.26

### 4.3 SphCNN vs. fourth-order tensor models

We compared the Frobenius Error of the predictions from the three SphCNN models with the fourth-order tensor models proposed by Moreno et al. (2016). Specifically, we computed the fourth-order fabric tensor models that use the mean intercept length (MIL) tensor, the generalized mean intercept tensor (GMIL), and the global structure tensor (GST). Table 4, Table 5 show the errors from models trained and evaluated on samples from the femur and for the whole dataset, respectively. Notice that the methods based on fourth-order fabric tensors perform much worse on the entire dataset. This might be attributed to two factors: first, the methods from Moreno et al. (2016) were tuned only with femur data, and second, the samples from other sites might be more challenging. Regarding the SphCNN models, while the accuracy of the best performing SphCNN is worse than the fourth-order GMIL, GST and the MIL tensor models for the femur samples, the error is more than 30% lower than the fourth-order models when the whole dataset is considered. That means that SphCNNs appears to be more robust with respect to the skeletal site.

**Table 4:** Mean and standard deviation (in parenthesis) of the Frobenius error computed on different cross-validation folds on the femur. The best average performance (i.e., lowest observed errors) is highlighted in bold.

Fold	GMIL	MIL	GST	SphCNN	SphCNN	SphCNN
				EGI	Channels	Scaling
1	0.17 (0.07)	**0**.**15** (**0**.**07**)	0.21 (0.09)	0.32 (0.24)	0.21 (0.13)	0.19 (0.10)
2	0.17 (0.06)	**0**.**15** (**0**.**07**)	0.22 (0.10)	0.26 (0.12)	0.27 (0.14)	0.26 (0.12)
3	0.15 (0.06)	**0**.**13** (**0**.**07**)	0.20 (0.10)	0.28 (0.12)	0.25 (0.15)	0.25 (0.14)
4	0.16 (0.07)	**0**.**14** (**0**.**07**)	0.21 (0.09)	0.27 (0.11)	0.29 (0.14)	0.28 (0.14)
5	0.17 (0.07)	**0**.**15** (**0**.**07**)	0.22 (0.10)	0.30 (0.15)	0.24 (0.14)	0.24 (0.14)

**Table 5:** Mean and standard deviation (in parenthesis) of the Frobenius error computed on different cross-validation folds on the combined dataset (femur, vertebra and radius). The best average performance (i.e., lowest observed errors) is highlighted in bold.

Fold	GMIL	MIL	GST	SphCNN	SphCNN	SphCNN
				EGI	Channels	Scaling
1	0.54 (0.30)	0.54 (0.29)	0.58 (0.30)	0.34 (0.36)	0.22 (0.14)	**0**.**22** (**0**.**13**)
2	0.48 (0.22)	0.48 (0.22)	0.52 (0.22)	0.29 (0.21)	**0**.**24** (**0**.**16**)	0.24 (0.17)
3	0.46 (0.20)	0.46 (0.20)	0.50 (0.20)	0.32 (0.26)	0.20 (0.14)	**0**.**19** (**0**.**15**)
4	0.46 (0.21)	0.47 (0.21)	0.50 (0.21)	0.28 (0.17)	**0**.**23** (**0**.**15**)	0.23 (0.22)
5	0.54 (0.27)	0.54 (0.27)	0.57 (0.27)	0.36 (0.36)	0.25 (0.21)	**0**.**23** (**0**.**16**)

Notice that the results reported in Moreno et al. (2016) are slightly different of the ones reported in Table 4. Unlike in that paper, only 80% of the femur samples are used to tune the parameters of the fourth-order tensor models in every fold. This is done to keep the consistency with the 5-fold cross-validation performed in this paper.

### 4.4 Skeletal site variability

In this section, we investigate the effect of samples taken from different bone regions on the resulting accuracy of the stiffness predictions. We trained multiple versions of the model that only inputs the EGI on different subsets of the training data. Each of those subsets contains only samples from a specific region, i.e., from the femur, radius, vertebra, or a combination of all of them. As before, we evaluated the resulting predictors on a hold-out set from the respective region with 5-fold cross-validation. In this experiment, we only used the EGI of the segmented image as the input of the SphCNN.

As shown in Table 6, all models performed far better on regions that were used for training compared to other regions. This suggests that the dataset seems to be quite heterogeneous with respect to the different regions. Therefore, it becomes difficult for the neural networks to predict stiffness tensors of trabecular bone of unseen skeletal sites. When all sites are used for training, the observed Frobenius error was similar for all regions. This indicates that the neural networks can generalize well to all data regions but only if they have seen them during training.

**Table 6:** Mean Frobenius Errors of SphCNN-based predictions trained and evaluated on subsets from different bone regions (all, radius, vertebra, femur). For each of the regions, we performed 5-fold cross-validation and ensured that samples only appeared in training or validation exclusively. Note also that if a sample from a specific site (e.g., the radius) was used in the training with ‘all’ samples, this sample was excluded from the ‘all’ and the specific (i.e., ‘radius’ in the example) evaluation regions. The best average performance (i.e., lowest observed errors) is highlighted in bold.

		Evaluation region			
Train region	Fold	All	Radius	Vertebra	Femur
All	1	0.32	0.29	0.35	0.30
	2	0.29	0.20	0.28	0.32
	3	0.29	0.33	0.30	0.27
	4	0.28	0.31	0.29	0.28
	5	0.30	0.21	0.34	0.29
	Mean	**0.30**	0.27	0.31	0.29
Radius	1	1.17	0.28	1.54	0.74
	2	1.53	0.18	2.08	0.88
	3	1.62	0.20	2.10	1.07
	4	1.66	0.23	2.26	0.94
	5	1.15	0.29	1.44	0.81
	Mean	1.43	**0**.**24**	1.88	0.89
Vertebra	1	0.56	0.65	0.28	0.61
	2	0.56	0.72	0.34	0.58
	3	0.57	0.69	0.27	0.61
	4	0.57	0.69	0.31	0.60
	5	0.57	0.70	0.33	0.59
	Mean	0.57	0.69	**0**.**31**	0.60
Femur	1	1.75	1.40	2.07	0.26
	2	2.23	1.47	2.68	0.30
	3	1.70	1.71	1.91	0.29
	4	1.73	1.24	2.05	0.28
	5	1.98	2.23	2.18	0.28
	Mean	1.88	1.61	2.18	**0**.**28**

### 4.5 Rotational equivariance

The spherical convolutions utilized in the proposed deep learning model are equivariant to actions of *SO*(3) (i.e., 3D rotations). In this section, we investigate how this equivariance property translates to the complete prediction model. We performed a qualitative evaluation by analyzing one good (Table 7), one medium (Table 8), and one poorly (Table 9) performing sample from the test set to the SphCNN model that only inputs the EGI. In this experiment, we varied the orientation of the original volumetric data, and the corresponding observed stiffness tensor. We then fed the rotated sample to our prediction model and evaluated the resulting Frobenius error. The rotation of the stiffness tensors was performed as described in Mehrabadi and Cowin (1990).

**Table 7:**
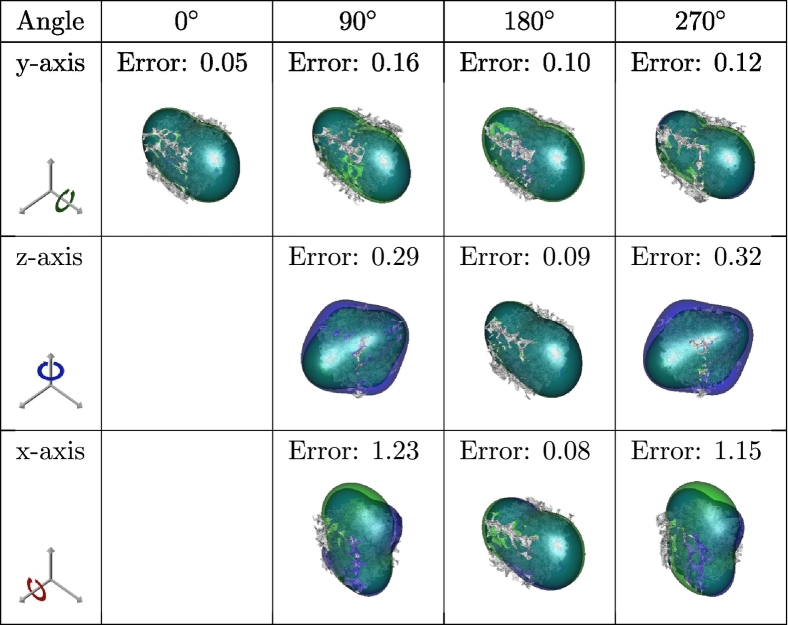
Reynolds-glyph visualizations of the observed (green) and predicted (blue) apparent stiffness tensors, superimposed over the respective bone segmentation (gray). The segmentation and the corresponding observed stiffness of a good-performing test sample (5% Frobenius error) were rotated according to the table headers. Then, the prediction was evaluated on the rotated variant to test the model's equivariance with respect to rotations. The Frobenius error between the observed and predicted stiffness is displayed over the visualizations.

**Table 8:**
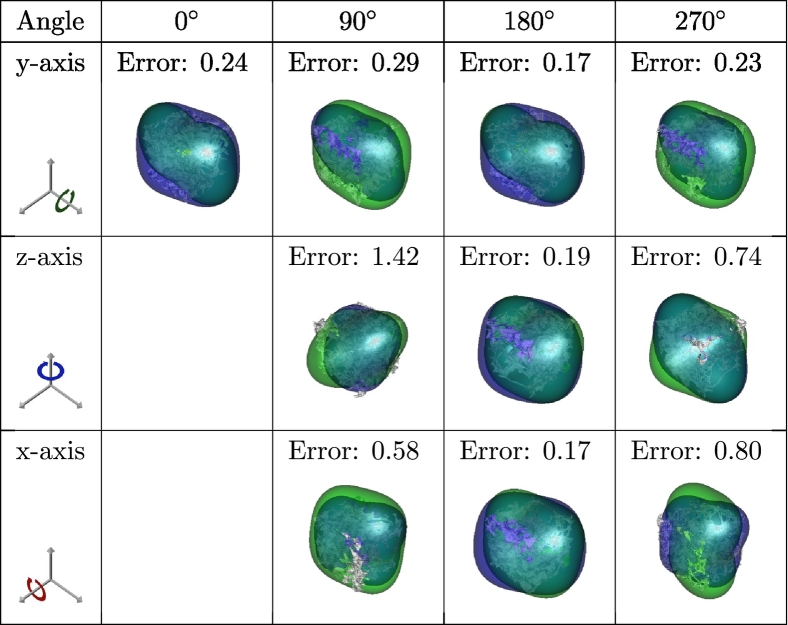
Similar visualizations as in Table 7 for a sample with an initial Frobenius error of 24%.

**Table 9:**
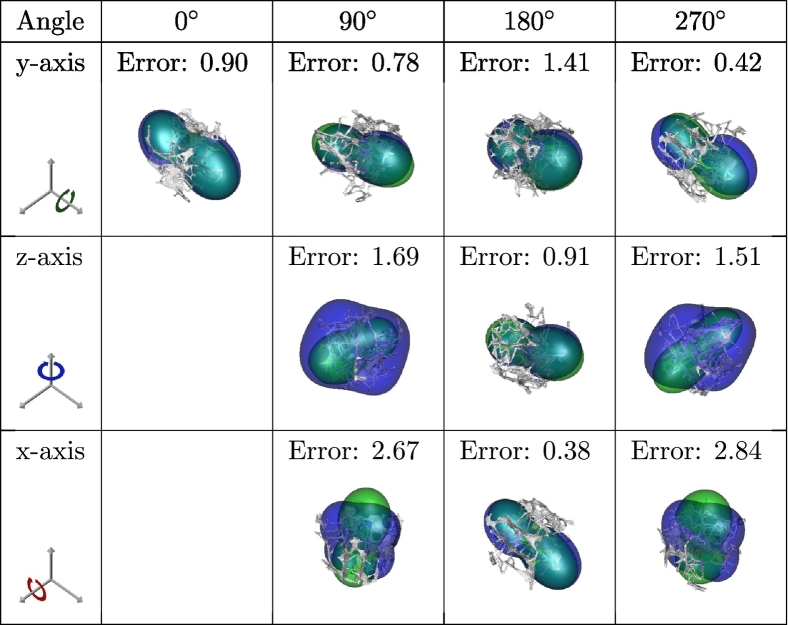
Similar visualizations as in Table 7 for a sample with an initial Frobenius error of 90%.

The first observation is that the complete prediction model is not equivariant to actions of SO(3) since different sample orientations resulted in various errors. Second, rotations around the y-axis seem to affect the prediction less than rotations around the other directions. A possible explanation for this effect might be related to how we represent the spherical EGI on a 2D grid. At some specific rotations, the presented method might lead to an oversampling of the data on the sphere's poles versus points closer to the equator. Therefore, a rotation aligned with the sphere's central axis (pole to pole) only translates the resulting EGI, while other rotations might perturb the data. It might be possible to tackle this problem by analyzing the poles of the EGI. In case much information of the EGI is concentrated on the poles, the procedure would be to estimate the EGI from the rotated image, predict the apparent stiffness tensor from the rotated image, and rotate back the prediction. It is, however, not obvious after which criteria the poles need to be analyzed (one possible criterion could be the occurrence of high-frequency signal components). Therefore, we leave this question open here for future research.

### 4.6 Bone density and Frobenius norm vs. Frobenius error

**Fig. 7:**
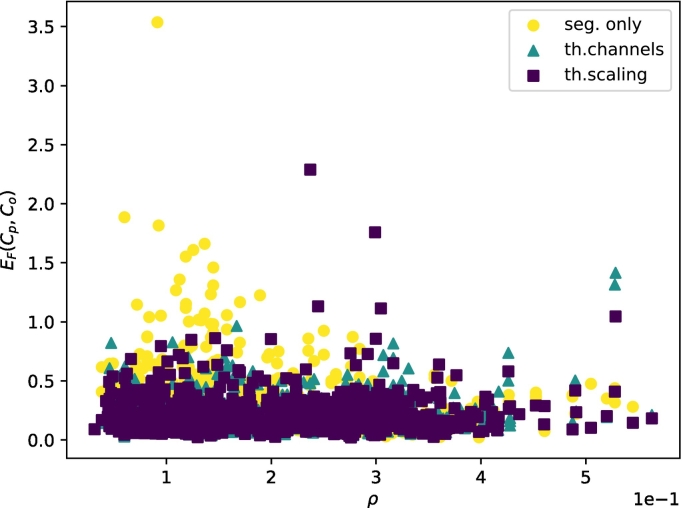
Correlation between the bone density *ρ* of the samples and the Frobenius error between the observed and predicted apparent stiffness tensors.

**Fig. 8:**
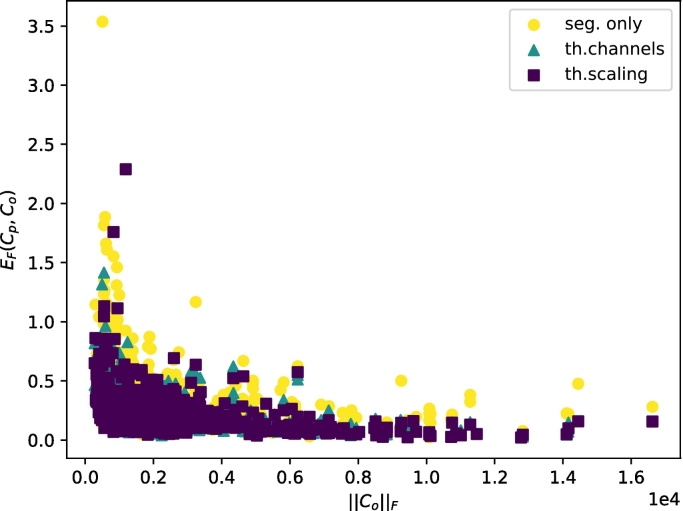
Correlation between the Frobenius norm of the observed stiffness tensor and the Frobenius Error between the observed and predicted apparent stiffness tensors.

Fig. 7, Fig. 8 show the relationship between the Frobenius error of the predictions and the bone density and Frobenius norm of the stiffness tensor, respectively. As shown, providing trabecular thickness and spacing information to the network helps to reduce the Frobenius error, especially at low bone densities. The same trend appears for the Frobenius norm of the stiffness tensor.

## 5 Discussion

In this work, the application of a DL-based pipeline to approximate complex volumetric FEM-based apparent stiffness predictions of trabecular bone samples was introduced. A significant constraint was the absence of a massive dataset, which is a requirement to train a deep 3D CNN from scratch. Instead, we projected our data into a reduced spherical domain and trained specific SphCNNs thereafter.

The experiments showed that the proposed spherical mapping is a reasonable candidate due to its low number of internal parameters. An additional advantage of the spherical mapping is that the method is able to estimate the apparent stiffness tensor independent of the imaging resolution or shape of the trabecular bone sample (e.g., cubes, cylinders, or spheres). Moreover, extensions of the method to in vivo acquisitions are feasible by using estimations of Tb.Th and Tb.Sp in grayscale (Moreno et al., 2012a). However, it is worth mentioning that a possible drawback of the spherical mapping and its subsequent dimensionality reduction is that it can also oversimplify the complexity of the trabecular bone structures. In order to tackle this issue, we proposed combining edge with thickness and spacing information and providing those as extra channels to the neural network. This approach led to a reduction of the overall error by around 11%. Using the EGIs computed from thickness and spacing maps was slightly better than using them for scaling the EGI computed from the segmented image. Unlike models based on fourth-order tensors, the trained neural networks are equally accurate for all tested skeletal sites. The best performing neural network reduced the Frobenius error of around 30% for the complete dataset compared to the fourth-order tensor models.

Our results also suggest that the trabecular bone at different sites might be heterogeneous. Thus, for our neural network to have a good performance, it is necessary to use training samples from all skeletal sites. The results also suggest that fourth-order tensor models might require a specific parameter tuning for every skeletal site and dataset. This is also true for other models (Zysset, 2003). In turn, such a tuning procedure is not needed with neural networks.

One specific theoretical aspect of stiffness tensor prediction is that it must be equivariant against *SO*(3) transformations (i.e., rotations in ℝ^3^). The accuracy of the prediction can be affected when the EGI contains much information close to the poles of the unitary sphere. The equivariance property can be used for estimating the stiffness tensor from a rotated (and more stable) version of the sample.

Finally, we found out that the prediction error is reduced when trabecular thickness and spacing information is added to the network, especially for specimens with low bone density. Still, the error is higher for specimens with a small Frobenius norm of the apparent stiffness tensor. This suggests that it is necessary to enhance the neural network's input with additional features that better capture the variability of the stiffness tensor in this case.

The best performing neural network was the one that only included the EGIs of the segmented image and the thickness map, with errors of around 20% regardless of the skeletal site. One possible reason to explain the better performance of this strategy compared to scaling the gradients is that, since the gradient of the segmented image is only non-zero in the surface of trabecular bone, thickness (or spacing) information is disregarded elsewhere in the scaling approach (cf. Fig. 4).

From the results, including thickness information both from the surface and inside the trabecular bone seems relevant for the estimation of stiffness. While this is true for thickness, only trabecular spacing information along the trabecular bone surface seems relevant for stiffness prediction since including spacing information from inside the marrow led to worse performance.

Unlike previous studies, the EGI was used instead of fabric tensors to model bone anisotropy (Zysset, 2003; Gross et al., 2013; Moreno et al., 2016). Notice that the EGI and fabric tensors are very related concepts. Indeed, the MIL, GMIL, and GST can be seen as filtered approximations of the EGI (Moreno et al., 2012b). Thus, using the EGI as the input of the neural network has the advantage that all available information related to the gradient of the image is used for the prediction.

Previous studies have also shown that bone density is relevant for stiffness predictions (Maquer et al., 2015). Our results align with these studies since almost all trained neural networks outperformed those that only used the EGI as input. It has been customary in previous methods to add the bone volume to total volume ratio (BV/TV) in models for predicting stiffness (Zysset, 2003; Gross et al., 2013; Moreno et al., 2016). Instead of using BV/TV, which is a global histomorphometric parameter, we used Tb.Th and Tb.Sp local maps. These maps can partially be seen as surrogates of local bone and local marrow content, respectively. With this approach, our goal was to expose the neural network to local bone and marrow content measures without the inherent loss of information that comes with the use of global parameters such as BV/TV, mean Tb.Th or mean Tb.Sp.

One of the main challenges of using DL in biomechanical applications is that this technology requires plenty of training data, which is usually unavailable (Ching et al., 2018). To solve this issue, Holzapfel et al. (2021) successfully combined advanced theoretical models with DL for characterizing mechanical properties of the arteries. In this paper, we dealt with that issue mainly by changing the domain of the data, in addition to data augmentation. Thus, these two studies show that it is possible to get the benefits of using DL in biomechanics if it is combined with additional strategies for dealing with the recurring problem of lack of data in biomechanical applications.

### 5.1 Future work

In addition to adding more local features to the neural networks, we plan to use other neural network architectures. Instead of SphCNNs, we plan to test graph CNNs (Wu et al., 2021), which might be useful for stiffness prediction. This may be possible by modeling trabecular bone as a graph. Further research avenues also aim to replicate the proposed process on larger datasets to verify the claims in terms of robustness and scalability.

## 6 Conclusion

In this paper, we propose the use of SphCNNs to approximate the apparent stiffness tensor of trabecular bone samples. The neural network makes use of local edge, thickness and spacing information to perform the prediction, which results in a reduction of more than 30% in the Frobenius error compared to state-of-the-art methods. Thus, SphCNNs are promising for replacing the more expensive μFE estimations.

## Funding

This work was partially supported by 10.13039/100013297Eurostars, grant E11626. We thank NVIDIA Corp. for the donation of the Titan Xp GPU we used in this study. The funding sources had no involvement in the research and preparation of this article.

## Consent for publication

All authors have given their consent for this publication.

## CRediT authorship contribution statement

**FS**: Conceptualization; Data curation; Formal analysis; Investigation; Methodology; Software; Validation; Visualization; Writing original draft. **JvK**: Methodology; Software; Writing - review & editing. **DHP**: Conceptualization; Data curation; Investigation; Methodology; Software; Validation; Writing - review & editing. **RM**: Conceptualization; Funding acquisition; Investigation; Methodology; Project administration; Resources; Software; Supervision; Validation; Writing - review & editing.

## Declaration of competing interest

The authors declare that they have no conflict of interest.

## Figures and Tables

**Table A.10:** Pairwise p-values with Bonferroni correction. The six methods were trained and evaluated on data from the femur site.

	SphCNN	EGI	SphCNN	GMIL	MIL	GST
	Channels		Scaling			
SphCNN channels		<0.0001	N.S.	<0.0001	<0.0001	<0.0001
EGI	<0.0001		<0.0001	<0.0001	<0.0001	<0.0001
SphCNN scaling	N.S.	<0.0001		<0.0001	<0.0001	<0.0001
GMIL	<0.0001	<0.0001	<0.0001		<0.0001	<0.0001
MIL	<0.0001	<0.0001	<0.0001	<0.0001		<0.0001
GST	<0.0001	<0.0001	<0.0001	<0.0001	<0.0001	

**Table A.11:** Pairwise p-values with Bonferroni correction. The ten methods were trained and evaluated on the complete dataset.

	SphCNN	SphCNN	SphCNN	SphCNN	SphCNN	SphCNN	SphCNN	GMIL	MIL	GST
	Channels	Channels	Channels	EGI	Scaling	Scaling	Scaling			
	(Th.Sp)		(Tb.Th)			(Tb.Sp)	(Tb.Th)			
SphCNN Channels (Th.Sp)		<0.0001	<0.0001	N.S.	<0.0001	<0.0001	<0.0001	<0.0001	<0.0001	<0.0001
SphCNN Channels	<0.0001		N.S.	<0.0001	N.S.	0.0001	N.S.	<0.0001	<0.0001	<0.0001
SphCNN Channels (Tb.Th)	<0.0001	N.S.		<0.0001	N.S.	<0.0001	<0.0001	<0.0001	<0.0001	<0.0001
SphCNN EGI	N.S.	<0.0001	<0.0001		<0.0001	N.S.	0.0004	<0.0001	<0.0001	<0.0001
SphCNN Scaling	<0.0001	N.S.	N.S.	<0.0001		<0.0001	<0.0001	<0.0001	<0.0001	<0.0001
SphCNN Scaling (Tb.Sp)	<0.0001	0.0001	<0.0001	N.S.	<0.0001		0.0013	<0.0001	<0.0001	<0.0001
SphCNN Scaling (Tb.Th)	<0.0001	N.S.	<0.0001	0.0004	<0.0001	0.0013		<0.0001	<0.0001	<0.0001
GMIL	<0.0001	<0.0001	<0.0001	<0.0001	<0.0001	<0.0001	<0.0001		N.S.	<0.0001
MIL	<0.0001	<0.0001	<0.0001	<0.0001	<0.0001	<0.0001	<0.0001	N.S.		<0.0001
GST	<0.0001	<0.0001	<0.0001	<0.0001	<0.0001	<0.0001	<0.0001	<0.0001	<0.0001	

## Appendix A Statistical comparison of the methods

The statistical significance of our results is accessed with pairwise Wilcoxon tests on all ten methods for the complete dataset and six methods for the femur. Here, we compare the distribution of the observed loss values from all folds. The *p*-values of the Table A.10, Table A.11 were corrected against multiple comparison by using Bonferroni's method.

## Footnotes

1 https://github.com/daniilidis-group/spherical-cnn.
